# Windows to the soul: vision science as a tool for studying biological mechanisms of information processing deficits in schizophrenia

**DOI:** 10.3389/fpsyg.2013.00681

**Published:** 2013-10-31

**Authors:** Jong H. Yoon, Summer L. Sheremata, Ariel Rokem, Michael A. Silver

**Affiliations:** ^1^Department of Psychiatry and Behavioral Sciences, Stanford University and Veterans Affairs Palo Alto Healthcare SystemPalo Alto, CA, USA; ^2^School of Optometry, Helen Wills Neuroscience Institute, and Vision Science Graduate Group, University of CaliforniaBerkeley, Berkeley, CA, USA; ^3^Department of Psychology, Stanford UniversityStanford, CA, USA

**Keywords:** schizophrenia, visual system, fMRI, psychophysics, magnetic resonance spectroscopy

## Abstract

Cognitive and information processing deficits are core features and important sources of disability in schizophrenia. Our understanding of the neural substrates of these deficits remains incomplete, in large part because the complexity of impairments in schizophrenia makes the identification of specific deficits very challenging. Vision science presents unique opportunities in this regard: many years of basic research have led to detailed characterization of relationships between structure and function in the early visual system and have produced sophisticated methods to quantify visual perception and characterize its neural substrates. We present a selective review of research that illustrates the opportunities for discovery provided by visual studies in schizophrenia. We highlight work that has been particularly effective in applying vision science methods to identify specific neural abnormalities underlying information processing deficits in schizophrenia. In addition, we describe studies that have utilized psychophysical experimental designs that mitigate generalized deficit confounds, thereby revealing specific visual impairments in schizophrenia. These studies contribute to accumulating evidence that early visual cortex is a useful experimental system for the study of local cortical circuit abnormalities in schizophrenia. The high degree of similarity across neocortical areas of neuronal subtypes and their patterns of connectivity suggests that insights obtained from the study of early visual cortex may be applicable to other brain regions. We conclude with a discussion of future studies that combine vision science and neuroimaging methods. These studies have the potential to address pressing questions in schizophrenia, including the dissociation of local circuit deficits vs. impairments in feedback modulation by cognitive processes such as spatial attention and working memory, and the relative contributions of glutamatergic and GABAergic deficits.

## Introduction

Schizophrenia is one of the most perplexing and important mysteries in modern medicine. This condition is associated with significant impairments across diverse functional domains, usually conferring to the affected individual a lifetime of disability and the need for long-term treatment. The prevalence of schizophrenia is nearly 1% of the general population, and it constitutes one of the largest public health burdens of any condition (Knapp et al., 2004). Among the diverse symptoms of schizophrenia, cognitive and information processing deficits are now widely recognized to be one of the most important causes of chronic functional impairments (Green et al., 2000). Unlike psychotic symptoms, deficits in cognition and information processing are largely refractory to currently available treatments (Goldberg et al., 2007). While intense research efforts have yielded tantalizing clues to the neural substrates of these deficits, we still possess limited understanding of the associated neural mechanisms. Further elucidation of these mechanisms will directly inform development of novel treatments. Thus, new approaches are needed to help spur advances in the discovery of neural mechanisms of cognitive and information processing deficits in schizophrenia.

The visual system has increasingly been recognized as having substantial advantages for conducting research on neural mechanisms of schizophrenia (Butler et al., 2008a; Green et al., 2009a; Silverstein and Keane, 2011a). Although visual disturbances are not generally considered to be among the most prominent clinical symptoms of schizophrenia, the visual system is nonetheless an important site of pathology and dysfunction in this disease. The high prevalence of visual perceptual abnormalities, including those occurring early in the course of illness, has been documented for many years by phenomenological studies (Chapman, 1966; Bracha et al., 1989). Moreover, a growing number of post-mortem studies suggest that these visual perceptual symptoms could be the result of neuropathologic abnormalities within the visual cortex (Selemon et al., 1995; Glantz and Lewis, 2000; Dorph-Petersen et al., 2007; Hashimoto et al., 2008). As others have previously noted (Silverstein and Keane, 2011b), the combination of the relatively advanced state of knowledge of visual physiology and functional anatomy compared to other brain systems and the availability of sophisticated investigative tools make vision an excellent system to investigate links among neural circuitry, information processing, and behavior. Thus, the study of visual processing abnormalities in schizophrenia presents rich opportunities to translate the tools, knowledge, and concepts from basic vision science to elucidate the mechanisms of impaired information processing in this condition.

These attributes of the visual system have resulted in an increasing number of investigations that have employed visual perceptual and neuroscience techniques to study schizophrenia. In the first part of this paper, we will review this emerging literature and highlight the confluence of factors that have made the visual system a useful model for the study of information processing deficits in schizophrenia. We have selected for review some of the most productive lines of research, including studies from our group, illustrating the diversity of visual processes that have been investigated and the range of paradigms that are available to study structure-function relationships and their perturbations in schizophrenia.

In the second part of this review, we highlight the many advantages of the visual system for translational research and describe some of the methods that allow inferences at a level of specificity that are typically not possible in studies of other brain systems. At the heart of these methods is psychophysics, the science of quantitatively measuring and modeling the relationships between physical properties of visual stimuli and their perceptual consequences. Psychophysical methods allow investigators to quantify and control for the generalized deficit confound in task performance in schizophrenia (Knight and Silverstein, 2001; Silverstein, 2008). The psychophysical study of perceptual phenomena can also lead to a deeper understanding of the computational constraints on the system, which in turn can illuminate the associated physiological mechanisms. Given these unique advantages of psychophysical approaches, we will review how they have been used to infer the physiological and computational mechanisms that underlie visual information processing.

Perceptual tasks can easily be paired with non-invasive functional neuroimaging, allowing testing and confirmation of many such inferences *in vivo*. After describing evidence of visual dysfunction in several domains of visual perception in schizophrenia, we will explain how psychophysical analysis and design elements of psychophysical tasks can be translated to the study of specific information processing deficits in schizophrenia.

We also discuss concepts that inform our thinking in considering the visual system as an *in vivo* model system for understanding information processing deficits in schizophrenia. These include the canonical cortical circuit: the conservation of the morphological and neurotransmitter subtypes of neurons and patterns of local connectivity across all of the neocortex, which suggests that findings from the visual system may generalize to other neocortical systems (Douglas and Martin, 2004). In addition, the conservation of specific aspects of the functional architecture of the visual system across species offers unique opportunities for translational neuroscience studies. Although schizophrenia affects a number of different neurophysiological and neurochemical systems, in this review we highlight the magnocellular pathway and the neurotransmitter GABA in visual processing. The visual studies that have been conducted in these systems illustrate the refined neurobiological inferences that may be drawn from schizophrenia studies of well-characterized systems in the brain.

Finally, in the last part of this review, we present a discussion of the ways in which visual studies may inform some of the most important unresolved questions relating to the pathophysiology of schizophrenia (glutamate and GABA abnormalities and local circuit vs. feedback modulatory deficits).

## Studies of visual perception in schizophrenia

Research in schizophrenia has examined a wide variety of processes that have implicated abnormalities at various levels of the visual system, from the retina (Balogh et al., 2008) to higher-order extrastriate visual cortex (Butler et al., 2008b). An exhaustive survey of visual studies in schizophrenia is beyond the scope of this paper. Instead, we review a select sample of studies, including ones conducted by our group, that illustrates the translational potential of the visual system in elucidating the underlying mechanisms of information processing deficits in schizophrenia. We emphasize two recurring themes shared by the visual studies reviewed here: the well-studied functional neuroanatomy of the visual system and the highly developed investigational tools that are available in the vision sciences. The first feature is important because it provides a relatively extensive knowledge base, compared to other neural systems, with which findings in schizophrenia may be contrasted to help identify abnormalities in this condition. Both features are critical in allowing investigators to test hypotheses and draw inferences at a level of specificity and neurobiological detail that is often not possible in other systems in the brain.

### Motion processing

Motion processing is among the most frequently studied visual processes in schizophrenia. Befitting such an ecologically critical function for primates, the primate brain has evolved a set of specific mechanisms for the processing of motion. Decades of basic research have identified a number of these mechanisms (Burr and Thompson, 2011), including the magnocellular visual processing stream that is specialized for detecting transient and dynamic changes in the visual environment (Livingstone and Hubel, 1988). Other brain regions involved in motion processing include visual cortical areas such as MT (medial temporal) and MST (medial superior temporal) that respond to moving stimuli and contain neurons with selectivity for motion features (Huk et al., 2002; Amano et al., 2009; Kolster et al., 2010). Note that the names of these areas refer to anatomical locations within the macaque brain, where they were initially discovered, and that homologous areas in the human brain are often referred to by the same names, even though these areas are not located in human medial temporal cortex. An extensive set of well-validated psychophysical methods for measuring components of motion processing has been established (Burr and Thompson, 2011), providing a foundation for the translation of motion processing paradigms into clinical studies.

Studying motion processing in patients with schizophrenia has proven important for several reasons. First, these studies show that visual paradigms can be used to reveal both impaired and preserved aspects of motion perception in schizophrenia, thereby providing a means of addressing the generalized deficit confound (Silverstein, 2008), a significant impediment to progress in schizophrenia research (see Overcoming the Generalized Deficit Confound section below). This confound reflects the fact that schizophrenia is characterized by deficits in almost any measured behavior (Knight and Silverstein, 2001). Thus, in the domain of motion perception, velocity discrimination (Chen, 2011) and spatial integration (Green et al., 2009a), but not direction discrimination (Chen et al., 2003), exhibit selective visual dysfunction in schizophrenia.

**Figure 1:**
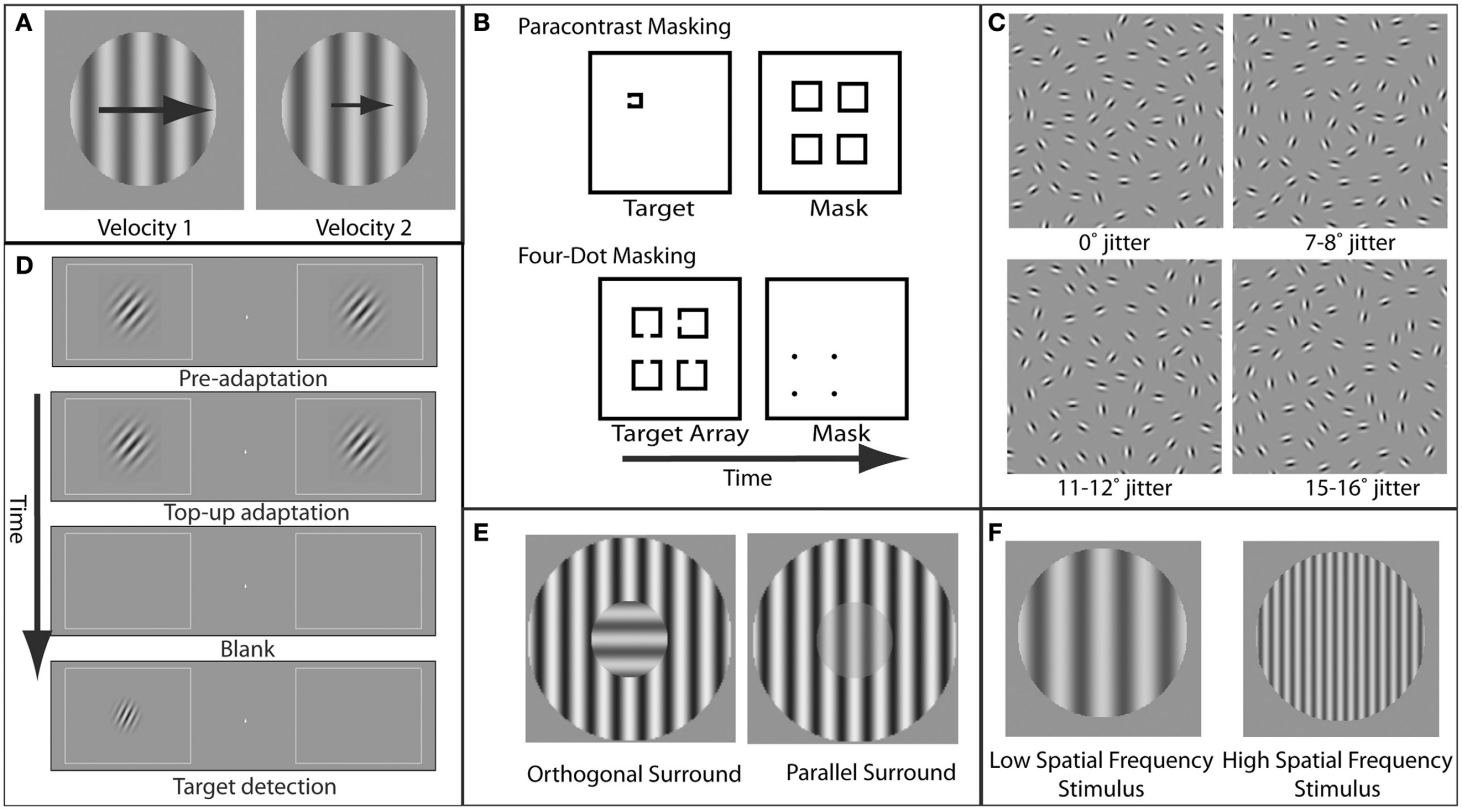
**Examples of paradigms and stimuli used to study specific visual deficits in schizophrenia. (A)** Velocity perception. In velocity discrimination tasks, subjects compare the velocity (represented here by the size of the arrows) of two sequentially presented stimuli (in this case, gratings). **(B)** Masking. In paracontrast masking, the mask interrupts formation of the stimulus percept. Because the mask does not spatially overlap the target contours, paracontrast masking cannot occur through integration of the target and mask. In four-dot masking, the mask is thought to disrupt reentrant processing but not formation of object representations. **(C)** Perceptual grouping. A set of elements in the display is perceptually grouped via contour integration to form a coherent percept (in this case, an ellipse). As the amount of orientation jitter between the different elements forming this shape is increased, the strength of the coherent percept decreases. **(D)** Orientation tuning. Adaptation to a grating with a specific orientation reduces the sensitivity for detection of a post-adaptation target grating with a similar orientation. The degree to which adaptation effects diminish with increasing adapter/target orientation differences provides an estimate of the width of orientation tuning in the visual system. **(E)** Orientation-specific surround suppression. The presence of a high-contrast surround decreases the perceived contrast of the center, and suppression is strongest when the orientation of the center and surround are parallel. **(F)** Magnocellular vs. parvocellular stimuli. Stimuli with low spatial frequencies preferentially engage the magnocellular pathway, while stimuli with higher spatial frequencies preferentially engage the parvocellular pathway.

Patients with schizophrenia and their first-degree relatives have long been known to have impairments in the ability to track moving objects with their eyes. Although these tracking eye movements, known as smooth pursuit eye movements, represent a relatively simple and common behavior, they nonetheless require highly precise coordination of visual and motor systems (Lisberger et al., 1987). Specifically, the successful tracking of an object first requires the ability to determine the velocity and direction in which the object is moving. While velocity discrimination (Figure 1A) is generally impaired in schizophrenia patients and correlates with eye tracking dysfunction (Chen et al., 1999), motion direction discrimination itself is not generally impaired and only demonstrates deficits when direction signals must be integrated over spatial locations (Chen et al., 2003). Motion processing deficits have also been found for higher-order motion in schizophrenia, even in the absence of lower-order motion processing deficits (Chen et al., 2004; Kandil et al., 2013).

Motion perception deficits have been reported not only in patients but also in their unaffected first-degree relatives (Chen et al., 2005). This suggests that motion perception deficits may have a genetic component and do not result from generalized deficits or medication effects. In addition, the fact that patients with schizophrenia have increased activity during motion processing in areas involved in cognitive control, such as the prefrontal cortex (Chen et al., 2008a), indicates that perceptual impairments may increase demand on cognitive control systems.

### Masking

Visual masking has been another rich area of research in schizophrenia. Masking refers to impaired perception of a behaviorally relevant stimulus (target) due to spatial and/or temporal proximity of a behaviorally irrelevant stimulus (mask) (Figure 1B). As extensively reviewed elsewhere (Green et al., 2011a), masking paradigms offer a number of advantages in elucidating neural mechanisms of information processing deficits in schizophrenia, including the ability to precisely control stimulus parameters, the presence of well established neurobiological models of masking, and the correlation of visual masking impairments with core symptoms in schizophrenia (Green and Walker, 1986; Braff, 1989).

While abnormalities in a variety of types of masking have been documented in schizophrenia, the best studied is backward masking. In backward masking, a mask is presented a fraction of a second after target onset. It is thought that two processes, interruption and integration, can contribute to visual masking and that these processes involve distinct neural mechanisms. Masking by interruption occurs after the target representation has already been formed and is based on interference with higher-level, feedback processes that underlie conscious perception of the target (Enns and Di Lollo, 2000). Masking by integration has been suggested to occur as a result of formation of a single perceptual representation of both the target and the mask (Turvey, 1973).

A recent study has employed the 4-dot masking paradigm, thought to selectively involve interference by interruption, to show that patients with schizophrenia show larger and more prolonged masking effects (Green et al., 2011b). In addition, unaffected first-degree relatives exhibit impaired performance on a backward masking task (Green et al., 1997). Patients with schizophrenia show abnormalities even when the target and mask do not overlap and masking by integration cannot occur (Green et al., 2011b), suggesting that abnormal masking is due to impairments in feedback processing. Electroencephalographic (EEG) studies have implicated mechanisms involving gamma-band synchrony in visual masking abnormalities in schizophrenia. In particular, patients with schizophrenia exhibited decreased evoked gamma-band activity during backward masking compared to controls but had intact gamma-band activity evoked by unmasked visual stimuli (Green et al., 2003; Wynn et al., 2005).

### Perceptual organization

Perceptual organization refers to the integration of visual elements across spatial locations to form a representation of a coherent object (Palmer, 1999) (Figure 1C). The idea that impairments in this integration may reflect underlying neural disorganization in schizophrenia has been the motivation for a number of studies (Phillips and Silverstein, 2003; Uhlhaas and Silverstein, 2005; Silverstein and Keane, 2011a). A variety of perceptual tasks have been used in these investigations, including viewing of luminance-binarized, or Mooney, faces (Uhlhaas et al., 2006a), identification of objects from fragmented images (Doniger et al., 2002; Sehatpour et al., 2010), perception of illusory contours (Spencer et al., 2003), and integration of interrupted contours (Silverstein et al., 2000; Uhlhaas et al., 2005, 2006b). There are strong associations between impairments in perceptual organization in schizophrenia and symptoms, including behavioral/cognitive disorganization (Silverstein et al., 2000; Phillips and Silverstein, 2003; Uhlhaas et al., 2005).

Object perception may require feature-binding processes that rely on neural synchrony (Engel et al., 2001). Patients with schizophrenia exhibited reduced phase synchrony in the beta (Uhlhaas et al., 2006a) and gamma (Spencer et al., 2003) bands during Gestalt perception. Moreover, patients with schizophrenia showed decreased BOLD responses to objects defined by contour integration in higher-order visual cortical areas (V2–V4) and in cortical attention networks but not in primary visual cortex (Silverstein et al., 2009). Similarly, when identifying an object from fragmented images, patients with schizophrenia exhibited decreased BOLD signal in visual and attention cortical networks, as well as the hippocampus, but not in primary visual cortex (Sehatpour et al., 2010). Because perceptual organization relies on visual processing areas beyond primary visual cortex (Fang et al., 2008), these findings suggest that perceptual organization deficits in schizophrenia do not result from impaired processing of individual visual elements but rather from abnormalities in specific early visual cortical circuits as well as in higher-order networks that modulate these circuits.

### Contextual modulation of visual processing

Contextual modulation in vision refers to the process by which perception or neural representation of a stimulus is influenced by the spatial and/or temporal context in which it is displayed (Albright and Stoner, 2002). Altered contextual modulation has been abundantly documented in schizophrenia using a wide variety of paradigms (Silverstein et al., 1996, 2013; Must et al., 2004; Dakin et al., 2005; Kéri et al., 2005; Tadin et al., 2006; Uhlhaas et al., 2006b; Chen et al., 2008b; Yoon et al., 2009; Schallmo et al., 2013; Tibber et al., 2013; Yang et al., 2013). The study of contextual modulation holds great promise for investigating the neural bases of impaired information processing in schizophrenia, as it arises from well characterized neural interactions that can be precisely manipulated by appropriate choices of stimuli and task parameters.

One of the best studied forms of contextual modulation in schizophrenia is surround suppression of contrast. This process refers to the reduction in perceived contrast of a stimulus when it is surrounded by a high-contrast stimulus (Chubb et al., 1989). Surround suppression is thought to play an important functional role in scene segmentation (Walker et al., 1999), enhancing salience processing (Petrov and McKee, 2006) and the generation of perceptual constancies (MacEvoy and Paradiso, 2001). Abnormal surround suppression has been reliably demonstrated in patients with schizophrenia across multiple tasks and stimuli (Dakin et al., 2005; Tadin et al., 2006; Chen et al., 2008b; Yoon et al., 2009; Tibber et al., 2013; Yang et al., 2013).

A specific form of surround suppression, referred to as orientation-specific surround suppression (OSSS) (Figure 1E), represents a particularly appealing paradigm for investigating schizophrenia. This appeal is due to the detailed and specific neurobiological inferences that can be made relating to this phenomenon. For oriented grating stimuli, surround suppression is greater when the target stimulus and the suppressive surround share the same orientation (parallel condition) (Cannon and Fullenkamp, 1991; Solomon et al., 1993; Xing and Heeger, 2000, 2001; Kosovicheva et al., 2012). This orientation specificity of surround suppression suggests a locus in early visual cortex, where neurons exhibit orientation-selective responses. Indeed, OSSS is also evident in the responses of single neurons in primary visual cortex (Blakemore and Tobin, 1972; Cavanaugh et al., 2002). Moreover, fMRI (Zenger-Landolt and Heeger, 2003) and magnetoencephalographic (MEG) and EEG (Haynes et al., 2003) correlates of surround suppression have been reported in human primary visual cortex. Furthermore, modeling of psychophysical and fMRI surround suppression data indicates that activity in visual cortical area V1 provides a better quantitative account of the behavioral results than higher-order areas (Zenger-Landolt and Heeger, 2003). This study is an example of the power of combining psychophysical measurements, functional imaging, and quantitative modeling to elucidate the neural mechanisms of a perceptual phenomenon.

OSSS has been measured perceptually with contrast discrimination thresholds for center stimuli in different surround orientations. Relative to a no-surround condition, healthy control subjects showed larger increases in contrast discrimination thresholds (i.e., more surround suppression) than patients with schizophrenia, and this group difference was selective for the parallel orientation condition (Yoon et al., 2009). Importantly, the finding of intact or even better performance in patients, compared to control subjects, mitigates the generalized deficit confound (Knight and Silverstein, 2001; Silverstein, 2008).

Two recent studies (Yang et al., 2013; Tibber et al., 2013) reported weakened surround suppression for contrast but not other features in patients with schizophrenia. The specificity of abnormalities in schizophrenia for contextual processing of contrast may explain why some studies have not found deficits in contextual modulation for other visual features in schizophrenia (Schütze et al., 2007; Roinishvili et al., 2008).

## Advantages of the visual system for translational research

### Conservation across species

The evolutionary preservation of functional characteristics of the visual system across mammals, such as orientation bandwidth of tuning functions in V1 and V2 (van den Bergh et al., 2010), provides motivation for studies of vision in translational research. Conservation of visual cortical organization across species allows translational conclusions to be made regarding neural circuits and molecular mechanisms from experiments in other mammalian species that cannot be conducted in humans. Molecular mechanisms of visual information processing can be studied using anatomical techniques, such as staining and visualization of particular molecular components in visual cortical circuits (Disney et al., 2007) and with pharmacological techniques that can be coupled with electrophysiological recordings of visual responses (Katzner et al., 2011).

Optogenetic studies allow selective stimulation of particular molecular components of visual circuits in a relatively temporally and spatially focal manner, thereby revealing links between molecular components and neural selectivity in the visual system. For example, GABAergic interneurons in the visual cortex are broadly divided into two different classes based on the relative expression of somatostatin and parvalbumin mRNA. In a rodent model, these two classes of cells have been associated with separate types of selectivity of responses to visual stimuli (Ma et al., 2010), and a recent study using optogenetic techniques (Lee et al., 2012) has provided causal evidence for this cell type-specific stimulus selectivity. Importantly, post-mortem studies have shown reduced expression of both somatostatin and parvalbumin mRNA transcripts in many cortical regions in patients with schizophrenia (Hashimoto et al., 2008).

In many cases, conservation of visual cortical and perceptual organization allows investigators to utilize similar or even identical brain measures and perceptual paradigms across species. Thus, the results of animal studies can directly inform human findings and strengthen neurobiological inferences. For example, OSSS has been studied in cats (Blakemore and Tobin, 1972) and non-human primates (Cavanaugh et al., 2002) to demonstrate surround suppression of responses of primary visual cortical neurons. Furthermore, pharmacological blockade of GABAergic receptors reduces surround suppression in primary visual cortex (Ozeki et al., 2004), while non-invasive measures of visual cortical GABA with magnetic resonance spectroscopy (MRS) is highly predictive of behavioral OSSS in humans (Yoon et al., 2010). Moreover, patients with schizophrenia exhibited lower behavioral OSSS as well as decreased visual cortical GABA levels (Yoon et al., 2010). Thus, the link between GABA and reduced OSSS in schizophrenia is significantly strengthened by the ability to investigate the same phenomenon (OSSS) using different experimental tools in a variety of species.

Synchronized oscillatory neural activity is another brain process with cross-species translational potential. In particular, oscillations in the gamma frequency range have been of particular interest, as they are considered to be a fundamental building block of neural computation subserving diverse functions [see Fries (2009) for review]. While a major focus of gamma-band synchrony research in schizophrenia has been higher-order cognition (Cho et al., 2006; Minzenberg et al., 2010), deficits in gamma-band synchrony have also been demonstrated for visual processes in schizophrenia (Spencer et al., 2003). The fact that the visual system has been one of the most active areas of gamma synchrony research in animal models (Singer and Gray, 1995; Womelsdorf et al., 2006; Bosman et al., 2012) provides many opportunities for translational research in this area.

### The canonical cortical circuit

Studying the visual system to gain fundamental insights about basic information processing deficits in schizophrenia is motivated by the fact that the neocortex has a high degree of conservation of morphological and neurotransmitter subtypes of neurons as well as patterns of laminar and tangential connectivity (Douglas and Martin, 2004). This cortical “canonical circuit” has been extensively characterized (Douglas et al., 1989; Markov et al., 2011) and includes laminar-specific reciprocal local connections between excitatory glutamatergic neurons and inhibitory GABAergic neurons, local excitatory/excitatory connections, and long-range excitatory inputs from, and outputs to, other cortical and subcortical regions.

Further evidence for the relevance of conservation of cortical circuits for information processing comes from modeling of responses in the frontal eye fields (FEF), a cortical area with neurons containing oculomotor signals. A model derived from detailed anatomical and physiological studies of early visual cortical area V1 was used to generate neuronal and connectivity parameters that were then applied to FEF, quantitatively accounting for responses of FEF neurons and FEF-dependent oculomotor behaviors in macaque (Heinzle et al., 2007) and patterns of eye movements of humans during reading (Heinzle et al., 2010).

In schizophrenia, there is significant and growing evidence that abnormalities in visual processing are correlated with core symptoms and functional impairments, including disorganization (Silverstein et al., 2000; Phillips and Silverstein, 2003; Uhlhaas et al., 2005), community functioning (Butler et al., 2005), and working memory (Haenschel et al., 2007). One interpretation of these findings, considered in the context of the canonical neocortical circuit, is that the visual cortical circuit abnormalities that presumably underlie visual processing deficits in schizophrenia reflect more general circuit abnormalities that are also present in higher-order neocortical areas, where they more directly affect disorganization and real-life functioning.

### Overcoming the generalized deficit confound

One of the main methodological concerns in the study of behavioral differences between patients with schizophrenia and healthy controls is the generalized deficit in performance that is found in practically all tasks. This deficit is attributable to impairments in a number of cognitive and/or affective processes (Heinrichs and Zakzanis, 1998). In studies aiming to focus on specific cognitive or sensory mechanisms, this generalized deficit is a particularly important but difficult confound that needs to be controlled (Knight and Silverstein, 2001).

**Figure 2:**
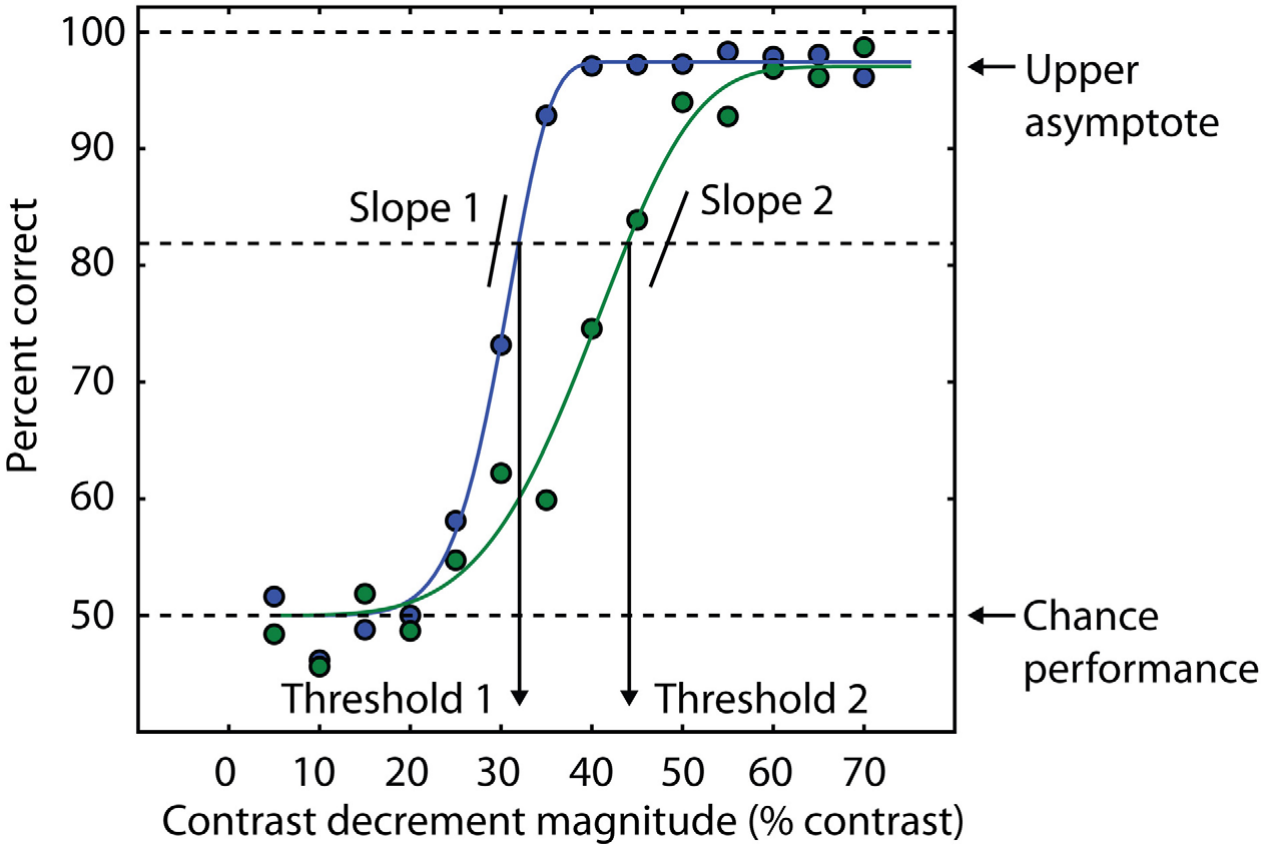
**Psychometric curves contain multiple sources of information about behavioral performance.** Behavioral performance, measured here as percent correct trials, is plotted as a function of a stimulus parameter (blue and green data points). In this example, the independent variable is the amount of decrement in contrast required to detect the presence of a target (see text and Yoon et al., 2009, 2010). Smaller contrast decrements are more difficult to discriminate, so the psychometric curve is monotonically increasing. In this example, cumulative Weibull functions are fit to the data (smooth green and blue lines). For each function, threshold is defined as the amount of contrast decrement that yields 82% correct trials. The percent correct corresponding to chance performance determines the lower asymptote of the curve (50% in this example). The blue curve has a lower threshold than the green curve, consistent with a higher percentage of correct trials at an equivalent contrast decrement compared to the green curve. Different slopes in the two curves indicate different levels of reliability across experimental conditions or groups of subjects. The upper asymptote is an index of attentional lapses, as the stimuli for these trials are well above perceptual threshold, and performance less than 100% indicates that attention was not properly engaged on some trials. The difference between the upper asymptote of the curve and 100% correct performance is a measure of the rate at which subjects are responding incorrectly on trials that should be easy to perform at 100% accuracy based on the physical level of the stimulus (e.g., large values of the contrast decrement in this task). This difference is comparable to estimates of the lapse rate in catch trials (e.g., Barch et al., 2012).

Fortunately, vision research provides several methods for addressing the generalized deficit confound, including several strategies that have been previously described (Silverstein, 2008). First, information available from psychometric functions can help isolate specific deficits in schizophrenia. The psychometric function is a model that relates behavioral performance on a task to a selected property of the physical stimulus (Figure 2). The slope of the psychometric curve indexes the reliability of the psychophysical threshold measurement (Macmillan and Creelman, 2004), and this fact has been used as a diagnostic tool for investigating visual disturbances in various patient groups (Patterson et al., 1980; Chauhan et al., 1993). More specifically, a shallow slope of the psychometric curve indicates an unreliable estimate of threshold due to inconsistent task performance. If group differences are found in this parameter, this may indicate a generalized deficit confound. On the other hand, the use of an upper asymptote parameter to fit models of psychometric data allows estimation of the frequency of lapses in attention that are not related to task difficulty (as determined by a physical stimulus feature). This is because the upper asymptote parameter of the curve quantifies subject performance when the stimulus is relatively easy to discriminate (Barch et al., 2012).

Quantitative modeling of the psychometric function can also generate confidence intervals for the values of the model parameters. One potential manifestation of a generalized deficit is that patients with schizophrenia might fatigue more easily over the course of an experimental session, resulting in deteriorating task performance. This would not only result in elevated thresholds in patients but also more variability in the threshold estimate, as the threshold would be changing during the session. This variability can be quantified with bootstrapping analysis (Efron and Tibshirani, 1993). We used psychometric functions to assess the possibility that generalized deficits affect the stability of OSSS performance in patients with schizophrenia (Yoon et al., 2009). We found no difference between patients and controls, suggesting that although generalized deficits may affect overall task performance (thresholds were generally higher in patients than in controls), they did not manifest as increased variability in performance throughout the experimental session. A similar analysis was also used in Dakin et al. (2005) and Tibber et al. (2013).

Importantly, many visual paradigms allow systematic manipulation of stimulus features in a way that does not affect the cognitive demands of the task. In the case of surround suppression, the generalized deficit confound can be addressed by varying the relative orientation of the surround and the center stimuli (Figure 1E). The task structure and demands do not differ between the parallel and orthogonal conditions, yet psychophysical thresholds are substantially different. Hence, an orientation-selective component of surround suppression can be isolated that is relatively independent of the cognitive demands of the task. This approach has revealed that although patients with schizophrenia have higher contrast discrimination thresholds, they show less OSSS (Yoon et al., 2009; see also Dakin et al., 2005; Tibber et al., 2013; Yang et al., 2013). In addition, patients' perception is more veridical regarding the actual contrast of the stimulus, relative to the biased (suppressed) perception in healthy controls (Dakin et al., 2005). It is hard to conceive how a generalized performance deficit would result in more accurate perception. These findings add to numerous studies that have utilized visual paradigms in which an abnormality in schizophrenia results in superior performance, compared to healthy subjects (Place and Gilmore, 1980; reviewed in Uhlhaas and Silverstein, 2005). We also note that the finding of reduced OSSS in schizophrenia (Yoon et al., 2009, 2010) presumably does not result from group differences in attentional lapses (Barch et al., 2012), as the frequency of these lapses would need to depend on the relative orientation of the center and surround stimuli to account for the observed differences between patients and control subjects.

Another example of the utility of visual psychophysics to address the generalized deficit confound in schizophrenia is that of Rokem et al. (2011). In this study, we employed an adaptation paradigm to measure selectivity for stimulus orientation in patients with schizophrenia and matched healthy controls (see Figure 1D). Participants viewed a high-contrast oriented adapting stimulus, followed by a post-adaptation probe in one of several different orientations relative to the adapter. Replicating previous results using this paradigm (Fang et al., 2005), we found that, for both patients and control subjects, adaptation elevated detection thresholds for probes having the same orientation as the adapter (0° offset). In addition, this elevation decreased systematically with increasing adapter-to-probe orientation offset, allowing the construction of a psychophysical tuning curve for orientation for each participant. To control for group differences in detection threshold that could have reflected generalized deficits, we fit a tuning curve model for each subject, normalized to that subject's threshold elevation at 0° offset. This procedure provides a measure of orientation tuning that does not depend on other aspects of performance such as cognitive control, attention, memory, etc. With this procedure, we found that subjects with schizophrenia have broader (less selective) orientation tuning. A similar approach could be adopted to explore other domains of selectivity for more complex stimuli (Schwarzlose et al., 2008).

### Magnocellular and parvocellular systems

The magnocellular processing stream is one of the most extensively studied visual pathways in schizophrenia and one that illustrates the refined neurobiological inferences that may be gained from study of the visual system. In the early stages of visual processing, it is thought that there are at least two distinct streams: magnocellular (M) and parvocellular (P) (Livingstone and Hubel, 1988; Felleman and Van Essen, 1991; Sincich and Horton, 2005). M neurons have relatively large receptive fields (i.e., they respond to a wide range of visual field locations) and therefore have low spatial resolution. These neurons respond best to coarse visual features (low spatial frequencies; Figure 1F), and are highly responsive even when stimuli have low luminance contrast. Furthermore, M neurons show strong responses to stimulus onsets and offsets and other visual transients. In contrast, P neurons have relatively small receptive fields and respond best to fine spatial details (high spatial frequencies; Figure 1F). P neuronal responses also require relatively high luminance contrast and are relatively insensitive to transient onsets of stimuli. M cells are thought to preferentially provide input to cortical areas that process motion (Livingstone and Hubel, 1988; but see Sincich and Horton, 2005).

The existence of these complementary processing streams allows investigators to create stimuli and conditions that are preferentially processed by either the M or P pathway. Using this approach, a series of studies has demonstrated selective processing deficits in the M pathway in schizophrenia. For example, Butler et al. (2001) found that patients with schizophrenia have decreased responses evoked by M-type stimuli, as measured with EEG. Furthermore, abnormal processing of M-type stimuli has been shown to contribute to motion processing deficits in schizophrenia (Kim et al., 2006). A recent fMRI study demonstrated a selective reduction in visual responses in the occipital cortex of patients with schizophrenia to contrast-reversing, low spatial frequency, sinusoidal gratings, especially at low contrast, consistent with a selective deficit in the M pathway (Martínez et al., 2008).

These findings suggest that further study of the M pathway could provide valuable information about the molecular and cellular bases of information processing deficits in schizophrenia. For example, some authors have interpreted the similarity between suppressed visual responses in subjects with schizophrenia and those of animals treated with NMDA receptor antagonists (Kwon et al., 1992) as indicating that NMDA receptor dysfunction may underlie impairments in feedforward signal amplification in schizophrenia (Butler et al., 2005). However, it should be noted that the M pathway deficit hypothesis is not universally accepted (Slaghuis, 1998; Kéri et al., 2002; Skottun and Skoyles, 2007). For example, masking deficits are associated with functional abnormalities within areas involved in object processing that are thought to receive preferential input from the P pathway (Green et al., 2009b). Specifically, in a backward masking paradigm, patients with schizophrenia exhibited decreased fMRI BOLD responses in lateral occipital cortex, an area involved in object identification, but normal responses in early visual cortical areas. This finding is consistent with other studies that have found visual masking deficits in schizophrenia that cannot be readily accounted for by M pathway deficits (Herzog et al., 2004; Chkonia et al., 2010).

## Future directions: GABA and glutamate deficits in schizophrenia

Some of the advantages of the visual system for schizophrenia research are illustrated by its potential utility in elucidating the relative contributions of inhibitory GABAergic and excitatory glutamatergic deficits in this condition. While multiple neurotransmitter systems are likely involved in schizophrenia, including dopamine (Davis et al., 1991), serotonin (Igbal and van Praag, 1995) and acetylcholine (Adams and Stevens, 2007), the GABA and glutamate systems are of particular interest because the interactions and balance of activity between these systems are fundamental phenomena underlying cortical information processing. Perturbation of this balance in schizophrenia may be a key pathophysiologic feature underlying cognitive and information processing deficits.

Post-mortem studies have consistently shown reductions in the concentration of the major synthetic enzyme for GABA within specific cortical interneuron subtypes, implying a deficit in GABAergic neurotransmission (Akbarian et al., 1995; Guidotti et al., 2000; Volk et al., 2000; Hashimoto et al., 2008). GABA release from these interneurons plays a critical role in controlling the activity of excitatory pyramidal neurons and in the generation of gamma-band synchrony (Whittington and Traub, 2003). As noted above, these coordinated oscillations are thought to be critical in supporting diverse processes, including higher-order cognition (Tallon-Baudry et al., 1998; Howard et al., 2003). However, the association between gamma-band synchrony and higher-order cognition can be confounded by gamma activity that is induced by microsaccades (Yuval-Greenberg et al., 2008). In schizophrenia, GABA deficits are thought to result in impairments in gamma-band synchrony, which in turn may be responsible for cognitive and information processing deficits (Lewis et al., 2005).

An alternative theory, the NMDA receptor hypofunction hypothesis, proposes that impairments in NMDA-type glutamate receptors are primary causal factors in schizophrenia. Specifically, it postulates that dysfunction of NMDA receptors on GABAergic interneurons leads to diminished GABAergic neurotransmission and, consequently, disinhibition and dysregulation of neural activity (Olney and Farber, 1995; Lewis and Moghaddam, 2006; Moghaddam and Javitt, 2012). Indirect supportive evidence for this hypothesis comes from animal studies in which induction of NMDA receptor hypofunction on GABAergic interneurons, either through pharmacologic (Homayoun and Moghaddam, 2007) or genetic (Belforte et al., 2010) manipulations in rodents, decreased excitatory drive onto GABAergic interneurons, and the resulting disinhibition caused an increase in spontaneous activity in excitatory pyramidal neurons.

In healthy humans, NMDA receptor antagonists such as PCP and ketamine can result in a behavioral syndrome that closely mimics the most salient clinical features of schizophrenia (Javitt and Zukin, 1991; Krystal et al., 1994). The disinhibition of excitatory activity that is hypothesized to occur in the initial stage of schizophrenia is thought to eventually lead to excitotoxic damage to cortical neurons and diminished excitatory drive (Olney and Farber, 1995). This may lead to compensatory homeostatic reductions in GABAergic tone in order to maintain the appropriate excitatory-inhibitory balance in cortical networks (Lewis et al., 2011). A more complete understanding of the relative contributions of glutamatergic and GABAergic deficits in schizophrenia would have obvious and important implications for the associated neural mechanisms of schizophrenia and for developing new interventions. However, as indicated above, there is extensive recurrent connectivity between excitatory and inhibitory neurons in cerebral cortex (for example, Ozeki et al., 2009), making it difficult if not impossible to manipulate activity in one system without eliciting secondary responses in the other.

### Direct MRS measurements of GABA and glutamate concentrations

Measurement of glutamate and GABA levels with MRS promises to be a powerful tool for dissociating the unique contribution of each neurochemical system in schizophrenia. This method allows non-invasive quantification of neurotransmitter levels in the brains of patients with schizophrenia, and unlike post-mortem studies, provides the opportunity to correlate measurements in the living brain with behavior in the same group of participants. Furthermore, MRS provides information about concentrations of neurotransmitters and other molecules in the native state, unaltered by pharmacological manipulations.

For the glutamate system, a large animal literature has documented activity-dependent changes in glutamate levels with invasive methods (Carder and Hendry, 1994; Qu et al., 2003), and MRS studies in humans have confirmed that dynamic increases in glutamate/glutamine (Glx) concentration occur in response to interventions designed to increase excitatory transmission (Mullins et al., 2005; Gussew et al., 2010; Maddock et al., 2011). Of direct relevance to visual system studies in schizophrenia is the recent demonstration at ultra high magnetic field that viewing of a contrast-reversing checkerboard increased visual cortical Glx concentration (Mangia et al., 2007).

Other studies have used MRS to document changes in GABA concentrations in response to cognitive, pharmacological, or other manipulations targeting the GABA system. For example, administration of vigabatrin, an inhibitor of GABA metabolism that increases GABA levels and is used in the treatment of epilepsy, caused dose-dependent changes in GABA levels (Verhoeff et al., 1999; Weber et al., 1999). In addition, motor cortical GABA MRS levels were reduced following a 30-min motor sequence learning paradigm thought to reduce tonic neural inhibition by decreasing GABA synthesis (Floyer-Lea et al., 2006). Finally, a number of studies have demonstrated strong correlations between GABA concentration and cognitive or perceptual functions (Edden et al., 2009; Sumner et al., 2010; Yoon et al., 2010; van Loon et al., 2013).

Taken together, these studies suggest that *in vivo* spectroscopic measurements of glutamate and GABA may index the underlying functional status of excitatory and inhibitory neural systems, respectively. Furthermore, the use of these non-invasive neuroimaging methods in conjunction with carefully selected visual paradigms and/or glutamatergic and GABAergic pharmacological agents can help dissociate excitatory and inhibitory deficits in schizophrenia. We employed this approach to document reduced visual cortical GABA levels as well as decreased behavioral OSSS in schizophrenia (Yoon et al., 2010). Across a sample of patients and control subjects, the amount of visual cortical GABA was highly predictive of OSSS, but no such correlation was observed for Glx and behavior (Yoon et al., 2010). Another application of GABA MRS comes from the study of receptor tyrosine kinase ErbB4, which is only expressed in GABAergic interneurons. A genetic variant of ErbB4 that is a risk factor for schizophrenia was shown to predict cortical GABA but not Glx concentrations (Marenco et al., 2011).

Despite the many advantages of MRS, the method is still being actively developed, and many issues remain unresolved. These include contamination of the measurement by other molecules and difficulties in obtaining reliable quantitative measurements of GABA concentrations, due to variability in GABA signal isolation as well as variability between individuals and between brain locations in the molecules used as baseline for comparison (see Mullins et al., 2013, for a recent review of these issues).

## Future directions: local circuit and feedback modulation deficits in schizophrenia

The visual system affords distinct advantages for experimental dissociation of local cortical circuit processing and feedback modulatory inputs from higher-order regions. In particular, manipulations of visual spatial attention allow comparison of neural responses to the identical visual stimulus when it is attended and when attention is directed elsewhere. Modulation of visual responses by spatial attention in healthy individuals has been shown in early visual cortex with fMRI (Gandhi et al., 1999; Somers et al., 1999), EEG (Martínez et al., 1999; Kelly et al., 2008), and MEG (Poghosyan and Ioannides, 2008). Moreover, sustained allocation of visual spatial attention to specific visual field locations increases fMRI signals in early visual cortical regions representing the attended locations, even in the absence of a visual stimulus (Kastner et al., 1999; Silver et al., 2007), thereby allowing a pure measure of top-down signals without any contribution from responses evoked by sensory stimulation.

**Figure 3:**
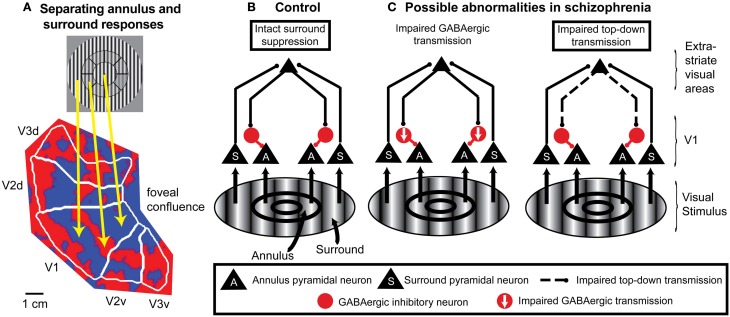
**An example of application of fMRI and a spatial attention paradigm to study impaired neural mechanisms of surround suppression in schizophrenia. (A)** Functional MRI data displayed on a flattened occipital cortical surface from an example subject. The circular grating stimulus is divided into two regions: the annulus and the “surround” (in this example, the “surround” includes visual field locations both inside and outside the annulus). An annulus was used as the “center” stimulus instead of a central circle or patch because it is difficult to definitively map retinotopic early visual cortical areas with fMRI for locations close to the fovea (central vision; where the gaze is directed). In these localizer data, subjects viewed the annulus stimulus in block alternation with the surround stimulus. The fact that early visual cortical areas (in this example, V1, V2, and V3) contain retinotopic maps of the visual field means that the responses to the annulus and surround portions of the stimulus are spatially separated on the cortical surface. Red indicates regions that exhibited a stronger response to the annulus than to the surround, and blue indicates regions that responded more to the surround than to the annulus. The foveal confluence is where the visual field maps in areas V1, V2, and V3 converge. Isoeccentricity lines form concentric arcs around the foveal confluence. This type of localizer experiment can be used to objectively define regions of interest in each visual cortical area in each subject that represent either the annulus or surround. **(B)** Neural circuit model of surround suppression of responses to grating stimuli in cortical area V1. Neurons representing the annulus or surround are largely spatially segregated within the V1 retinotopic map but project to common target neurons in higher-order visual cortical areas. These areas send feedback projections back to V1 that activate local GABAergic inhibitory neurons. The resulting inhibition suppresses responses in V1 neurons that represent annulus locations. **(C)** Possible circuit-level abnormalities contributing to reduced surround suppression in schizophrenia. Left, reduction in the number and/or efficacy of GABAergic neurons within V1 could decrease inhibition of the pyramidal neurons. Right, impaired feedback modulation to V1 could result in reduced excitation of GABAergic interneurons. Experimental manipulations of spatial attention can help distinguish between these possible mechanisms.

Early visual cortical areas contain a continuous topographic map of visual field locations on the cortical surface. That is, a visual stimulus presented at a particular visual field location activates a corresponding location in each of these cortical areas, and the layout of visual field locations reflects the two-dimensional representation of visual field locations in the retina (retinotopic maps; Figure 3A). More than 20 distinct cortical areas have now been identified in the human brain based on their topographically-organized visual field maps (Silver and Kastner, 2009; Wandell and Winawer, 2011).

The existence of topographically-organized visual field maps in the visual system, combined with appropriate manipulations of spatial attention, allows fMRI responses to attended and unattended visual field locations to be simultaneously measured in independent sets of voxels within a given cortical area. Feedback modulation of fMRI responses in early visual cortex by spatial attention is spatially-specific: it is restricted to those portions of the topographic visual field maps that represent the attended portions of the visual field (Tootell et al., 1998; Silver et al., 2007). One potential source of these top-down spatial attention signals is intraparietal sulcus areas such as IPS1 and IPS2 (Silver et al., 2005). These areas contain topographic maps of the locus of spatial attention and transmit spatially-selective top-down attention signals to early visual cortex (Lauritzen et al., 2009; Greenberg et al., 2012).

The utility of using spatial attention manipulations to separately and simultaneously measure responses to attended and unattended stimuli can be extended to other paradigms in which stimuli are presented in different visual field locations. For example, attention manipulations can be included in fMRI studies of surround suppression that retinotopically identify portions of visual cortical areas that represent visual field locations corresponding to either the center or surround (Figure 3A).

The combination of OSSS and spatial attention holds great promise in characterizing local circuit vs. feedback modulation abnormalities in schizophrenia. The fact that OSSS is selective for stimulus orientation suggests an early visual cortical locus, and finding a neural correlate of reduced OSSS in early visual cortex in schizophrenia would establish an excellent experimental model in a portion of the brain that, relative to other brain regions, is well understood.

In addition, comparison of responses to the suppressed center stimulus when it is attended vs. when attention is drawn elsewhere (for example, maintaining attention at central fixation by requiring subjects to perform a difficult task there) provides a highly controlled measure of feedback modulation of visual cortical activity by spatial attention in schizophrenia. The use of fMRI or other neurophysiological techniques uniquely allow measurement of responses to a stimulus when it is being ignored. Analogous measures of the impact of unattended stimuli are very difficult to obtain with behavioral methods, because requiring a subject to make a behavioral response to a stimulus necessarily requires the allocation of some attentional resources (typically an unknown amount) to that stimulus.

Finally, combining spatial attention manipulations with OSSS is likely to provide insights regarding the causes of reduced OSSS in schizophrenia at the local cortical circuit level. For grating stimuli, the neural substrates of surround suppression are thought to include feedback projections from higher-order visual cortical areas to area V1 (Angelucci and Bressloff, 2006; Nassi et al., 2013), with these feedback connections suppressing V1 pyramidal cell responses through local GABAergic interneurons (Ozeki et al., 2004, 2009; Schwabe et al., 2006) (Figure 3B). In this framework, reduced OSSS in schizophrenia could be due to impaired feedback modulation of V1 and/or diminished GABAergic transmission (Yoon et al., 2010) (Figure 3C). Spatial attention is a well-controlled and thoroughly studied means of manipulating top-down inputs, and single-unit studies indicate that directing spatial attention to the center vs. surround modulates surround suppression of neural responses in cortical area V4 (Sundberg et al., 2009). In fMRI studies of schizophrenia, finding reduced OSSS that is independent of whether the center stimulus is attended or not would be evidence of a local cortical circuit mechanism. On the other hand, if reduced OSSS in schizophrenia is only evident when the center stimulus is attended, this would implicate feedback modulation by spatial attention.

## Conclusion

While contributions from multiple disciplines and experimental approaches will likely be required to overcome the formidable challenges in elucidating the neural mechanisms of cognitive and information processing deficits in schizophrenia, the study of the visual system has a number of distinct advantages in this area. This review has highlighted some of the most productive lines of research within the rapidly growing body of literature on visual processing in schizophrenia, illustrating the diversity of visual processes that have been studied as well as the sophisticated methods available in the vision sciences. We also discussed several key factors that make the visual system such an appealing model system for the discovery of neural mechanisms. The convergence of the well-developed body of knowledge in structure-function relationships in the visual system, the conservation of the functional architecture across species, the preservation of basic local circuit architecture across neocortical regions, and the availability of quantitative methods to control for generalized deficits allow for inferences at a level of detail and specificity that is usually impossible in other neural systems. In the near future, the combination of vision science paradigms with modern neuroimaging methods may allow us to test some of the most compelling hypotheses on the neural origins of cognitive and information processing deficits in schizophrenia.

### Conflict of interest statement

The authors declare that the research was conducted in the absence of any commercial or financial relationships that could be construed as a potential conflict of interest.
